# Supplementary figures and images for: De novo identification of satellite DNAs in the sequenced genomes of Drosophila virilis and D. americana using the RepeatExplorer and TAREAN pipelines
Source: PLoS One. 2019 Dec 19;14(12):e0223466. doi: 10.1371/journal.pone.0223466 (PMC6922343; doi:10.1371/journal.pone.0223466)

**a****859692 read total**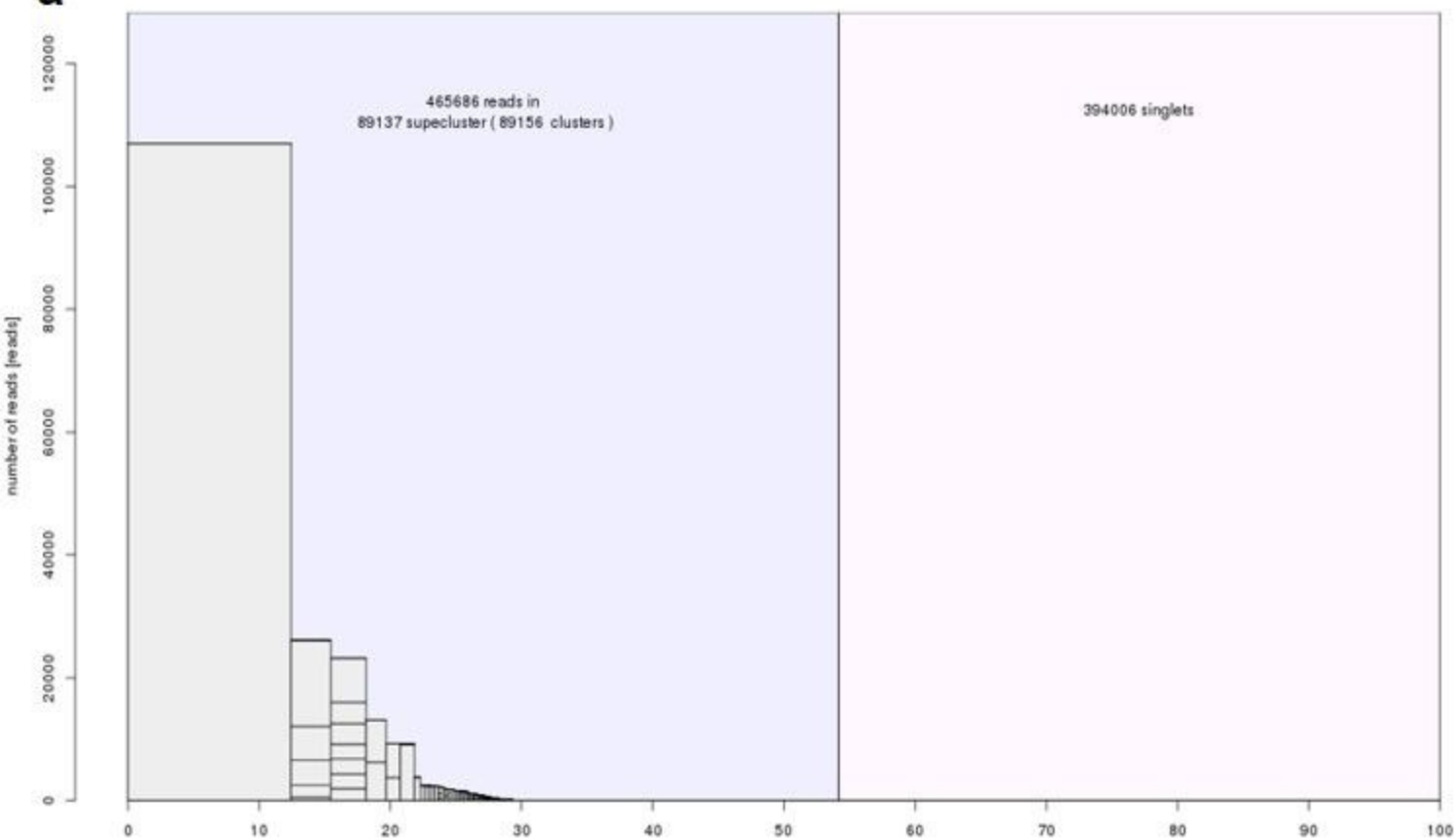**b****1219656 read total**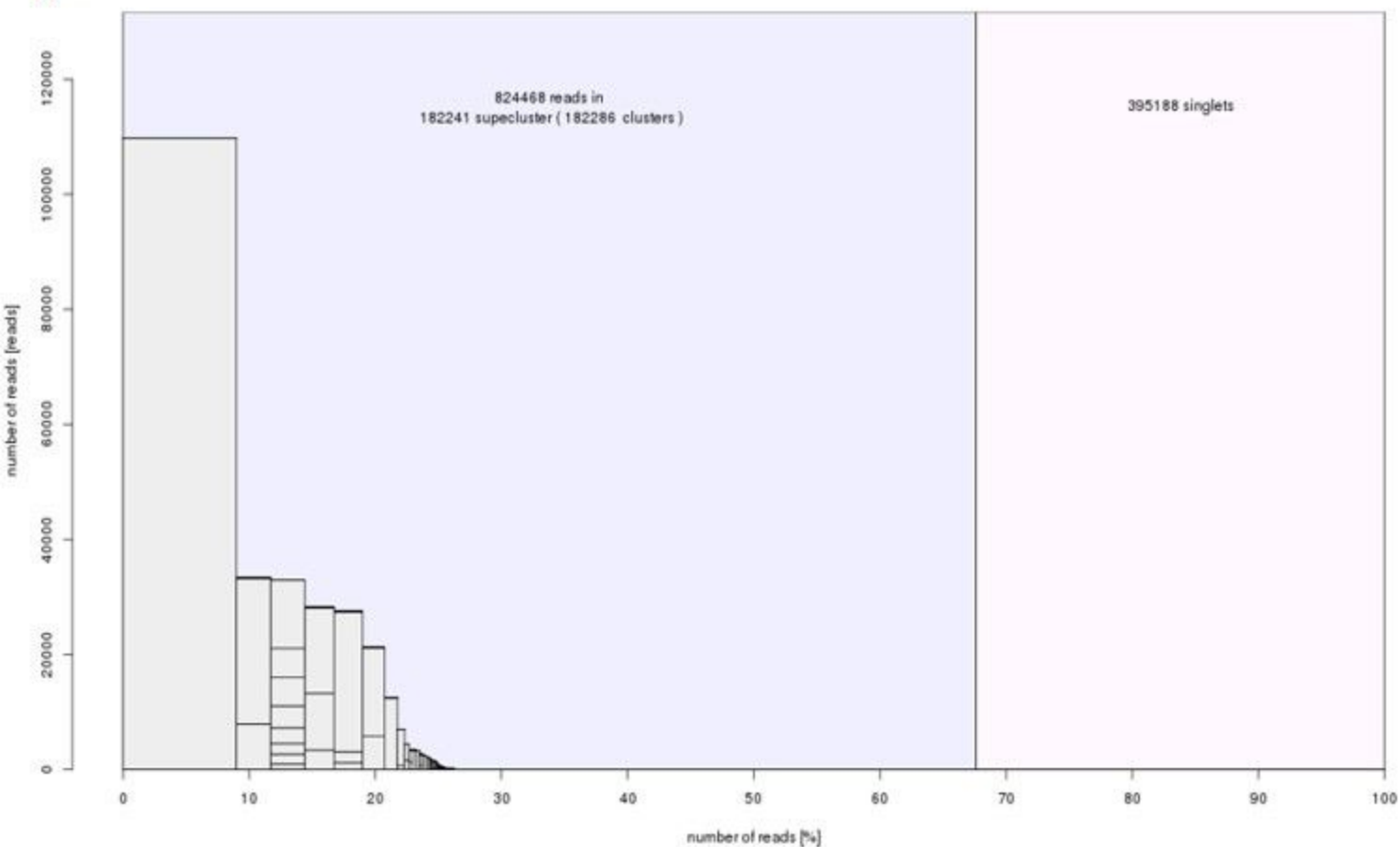

Supplement: S1 Fig — The histogram analysis is the overall result of the clustering process, after filtering and pre-processing of raw reads. It shows (on the top), the total number of reads analyzed during the run. Each column represents a cluster (by abundance from left to right). The y-axis refers to the number of reads by cluster and the x-axis the percentage of each cluster in the analysis. (PDF) [file pone.0223466.s001.pdf]

CL5  
6881

■ NAN

CL6  
6233

CL5 ----> CL6

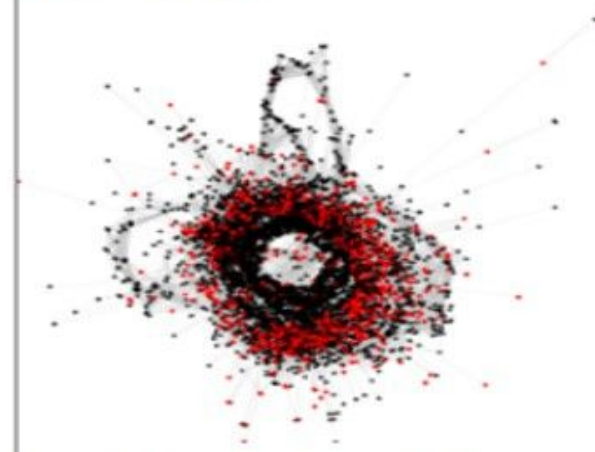

No. of shared pairs: :1242

CL6 ----> CL5

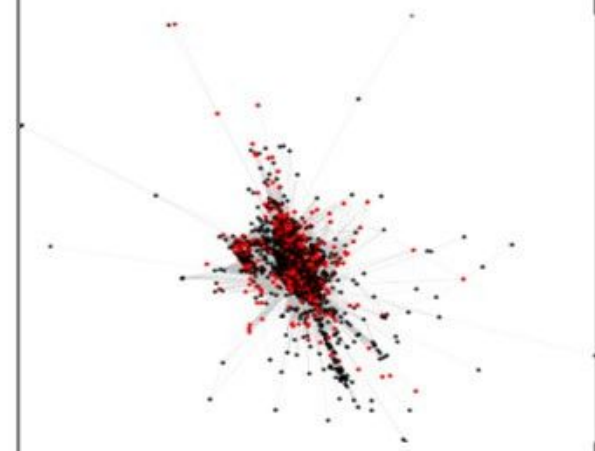

No. of shared pairs: :1242

Supplement: S2 Fig — (PDF) [file pone.0223466.s002.pdf]

CL4  
15385

■ NAN

CL11  
5811

CL4 ----> CL11

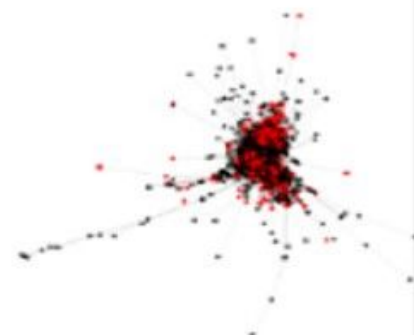

No. of shared pairs: :2795

CL11 ----> CL4

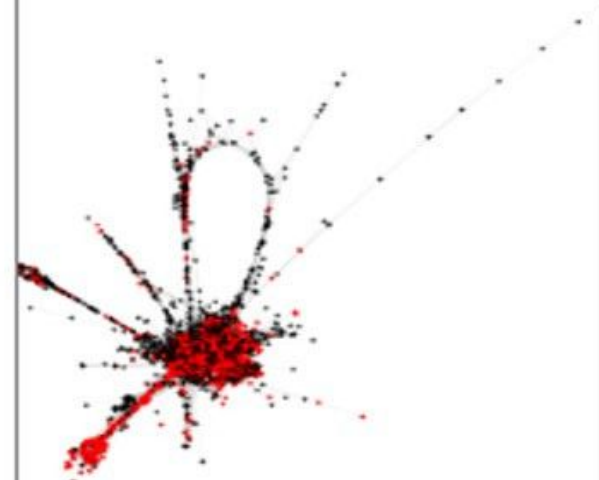

No. of shared pairs: :2795

Supplement: S3 Fig — (PDF) [file pone.0223466.s003.pdf]
