# Supplementary material for: De novo identification of satellite DNAs in the sequenced genomes of Drosophila virilis and D. americana using the RepeatExplorer and TAREAN pipelines
Source: PLoS One. 2019 Dec 19;14(12):e0223466. doi: 10.1371/journal.pone.0223466 (PMC6922343; doi:10.1371/journal.pone.0223466)

Cluster no. 1

Go back to cluster table

Cluster is part of supercluster: 1

Cluster characteristics:

size	40000
size_real	106913
ecount	890629
supercluster	1
annotations_summary	0.00% Class_I/LTR/Ty3_gypsy:Ty3-INT
pair_completeness	0.926082726814153
pbs_score	0
TR_score	0.866992134831461
TR_monomer_length	7
loop_index	0.967241119429232
satellite_probability	0.819019943211142
consensus	TTTGTAG
TAREAN_annotation	Putative satellite (high confidence)
orientation_score	0.999951596208121

Reads annotation summary

	cl_string	domain	Freq	proportion
Ty3_gypsy Ty3-INT	Ty3_gypsy Ty3-INT		1	9.4e-06

clusters with similarity:

Cluster	Number of similarity hits
39	2
43	2

clusters connected through mates:

Cluster	Number of shared read pairs	k
2	64	0.0198
3	31	0.013
4	31	0.0135
8	29	0.00672
5	26	0.00865
7	24	0.00897
11	22	0.00872
6	19	0.0063
13	19	0.00805

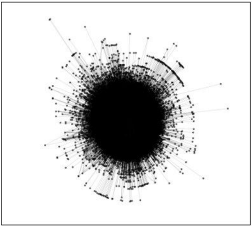

Supplement: S4 Fig — (PDF) [file pone.0223466.s004.pdf]
