# Supplementary material for: De novo identification of satellite DNAs in the sequenced genomes of Drosophila virilis and D. americana using the RepeatExplorer and TAREAN pipelines
Source: PLoS One. 2019 Dec 19;14(12):e0223466. doi: 10.1371/journal.pone.0223466 (PMC6922343; doi:10.1371/journal.pone.0223466)

Cluster no. 1

Go back to cluster table

Cluster is part of supercluster: 1

Cluster characteristics:

size	40000
size_real	109744
ecount	953793
supercluster	1
annotations_summary	
pair_completeness	0.97573182587405
pbs_score	0
TR_score	0.894212222222222
TR_monomer_length	7
loop_index	0.964715639214898
satellite_probability	0.917376582447331
consensus	TACAAAC
TAREAN_annotation	Putative satellite (high confidence)
orientation_score	1

Reads annotation summary

No similarity hits to repeat databases found

clusters with similarity:

Cluster	Number of similarity hits
6	19
54	2
760	2

clusters connected through mates:

Cluster	Number of shared read pairs	k
6	168	0.0887
3	17	0.00682
2	16	0.00648
54	12	0.0162
11	11	0.00451
121	10	0.0144
77	9	0.0122
4	5	0.00209
76	5	0.00735

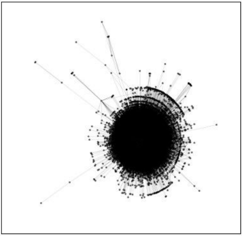

Supplement: S5 Fig — (PDF) [file pone.0223466.s005.pdf]
