# Supplementary material for: De novo identification of satellite DNAs in the sequenced genomes of Drosophila virilis and D. americana using the RepeatExplorer and TAREAN pipelines
Source: PLoS One. 2019 Dec 19;14(12):e0223466. doi: 10.1371/journal.pone.0223466 (PMC6922343; doi:10.1371/journal.pone.0223466)

Cluster no. 2

Go back to cluster table

Cluster is part of supercluster: 2

Cluster characteristics:

size	13980
size_real	13980
ecount	9974464
supercluster	2
annotations_summary	0.01% Class_I/LTR/Ty3_gypsy:Ty3-GAG<br>0.01% 45S_rDNA/25S_rDNA<br>0.01% Class_I/LTR/Ty3_gypsy:Ty3-INT
pair_completeness	0.709882583170254
pbs_score	0
TR_score	0.508430588235294
TR_monomer_length	154
loop_index	0.936552217453505
satellite_probability	0.0357308176144574
consensus	TTTTGTTTTTCAAGATATCTTGACCAAACTCGGCATTTATTAGTTTTACTATACTCCTCATATATATGCAAAATCCTATT<br>AAGATCGGACCACTATATCATATAGCTGCCATAGGAACGATCGGTCGAAAATTAAGTTTTTGTATGAAAAAACA
TAREAN_annotation	Putative satellite (low confidence)
orientation_score	1

Reads annotation summary

	cl_string	domain	Freq	proportion
25S_rDNA	25S_rDNA		1	7.2e-05
Ty3_gypsy Ty3-GAG	Ty3_gypsy	Ty3-GAG	1	7.2e-05
Ty3_gypsy Ty3-INT	Ty3_gypsy	Ty3-INT	1	7.2e-05

clusters with similarity:

Cluster	Number of similarity hits
9	5120
53	4550
94	727
89	618
187	16
27	13
2410	4
296	3
6	[illegible]

clusters connected through mates:

Cluster	Number of shared read pairs	k
9	467	0.149
53	183	0.126
89	94	0.0757
1	64	0.0198
94	53	0.0423
7	50	0.0276
11	44	0.0266
27	42	0.0279
10	37	0.0261

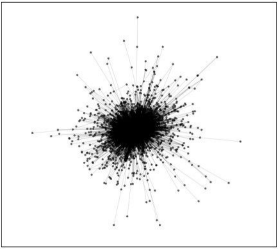

Supplement: S6 Fig — (PDF) [file pone.0223466.s006.pdf]
