# Supplementary material for: De novo identification of satellite DNAs in the sequenced genomes of Drosophila virilis and D. americana using the RepeatExplorer and TAREAN pipelines
Source: PLoS One. 2019 Dec 19;14(12):e0223466. doi: 10.1371/journal.pone.0223466 (PMC6922343; doi:10.1371/journal.pone.0223466)

Cluster no. 3

Go back to cluster table

Cluster is part of supercluster: 5

Cluster characteristics:

size	15038
size_real	24408
ecount	19830835
supercluster	5
annotations_summary	0.00% Class_I/LTR/Ty3_gypsy:Ty3-RT
pair_completeness	0.740444951511694
pbs_score	0
TR_score	0.506713953488372
TR_monomer_length	154
loop_index	0.961499966637753
satellite_probability	0.0439494278251884
consensus	TTAAGTTTTTGTATGAAAAAACATTTTGTTTATCAAGATATCTTGACCAAACTCGGCATTTATTAGTTTTACTATACTCC<br>TCATATATATGCAAAATCCTATTAAGATCGGACCACTATATCATATAGCTGCCATAGGAACGATCGGTCGAAAA
TAREAN_annotation	Putative satellite (low confidence)
orientation_score	0.999999949584211

Reads annotation summary

	cl_string	domain	Freq	proportion
Ty3_gypsy Ty3-RT	Ty3_gypsy	Ty3-RT	1	4.1e-05

clusters with similarity:

Cluster	Number of similarity hits
25	41500
32	5620
8	99
29	11
43	6
60	6
10	4
1410	3
4	2

clusters connected through mates:

Cluster	Number of shared read pairs	k
25	678	0.246
8	599	0.0904
32	402	0.167
5	108	0.0124
10	60	0.024
29	50	0.0216
15	31	0.00933
43	19	0.00976
1	17	0.00682

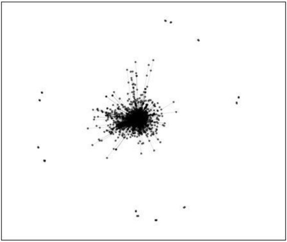

Supplement: S7 Fig — (PDF) [file pone.0223466.s007.pdf]
