# Supplementary material for: De novo identification of satellite DNAs in the sequenced genomes of Drosophila virilis and D. americana using the RepeatExplorer and TAREAN pipelines
Source: PLoS One. 2019 Dec 19;14(12):e0223466. doi: 10.1371/journal.pone.0223466 (PMC6922343; doi:10.1371/journal.pone.0223466)

Cluster no. 5

Go back to cluster table

Cluster is part of supercluster: 4

Cluster characteristics:

size	6881
size_real	6881
ecount	833175
supercluster	4
annotations_summary	
pair_completeness	0.565999089667729
pbs_score	0
TR_score	0.376863561660416
TR_monomer_length	370
loop_index	0.947284112421727
satellite_probability	0.0113560037017239
consensus	TGGCCGATTTGGGTCAAAATTTAGATTTTCTATTTTTTGATGCCAGTCGATAGTACTGATCCTTACGAGTCCAAAATGGT<br>ATAAAATTTGAAAATCGGACATTATTTGGCCGAGATATTCCAAAAAATCATAAGGAAAGGTTGACTTTACAAACGAATCA<br>TTTTCGGGTCCACATCTTTTTGTAACCCATCGATTTTTAATTTGTTTGAATGCCAATTGATAGTAGGGATCCGTACAAAT<br>TCAAAAAGGTATAATATTTGAAAATCAGACTTTATTTACCCGAGATATTAAAAAAAAATCATCAAAAAAGGTTTGATTTT<br>CGAACCGACCCCGATTTTGATAAAAAACGTGATGTAACCCCTTGCCATTT
TAREAN_annotation	Putative satellite (low confidence)
orientation_score	0.999979608360572

Reads annotation summary

No similarity hits to repeat databases found

clusters with similarity:

Cluster	Number of similarity hits
6	28100
26	46
87	26
9	6
24	5
8	2
73	2
2	1
46	1

clusters connected through mates:

Cluster	Number of shared read pairs	k
6	1240	0.648
87	53	0.0532
26	48	0.0456
8	37	0.0115
9	30	0.0104
24	28	0.0165
1	26	0.00865
2	12	0.00561
7	9	0.00571

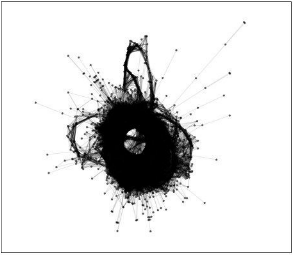

Supplement: S8 Fig — (PDF) [file pone.0223466.s008.pdf]
