# Supplementary material for: De novo identification of satellite DNAs in the sequenced genomes of Drosophila virilis and D. americana using the RepeatExplorer and TAREAN pipelines
Source: PLoS One. 2019 Dec 19;14(12):e0223466. doi: 10.1371/journal.pone.0223466 (PMC6922343; doi:10.1371/journal.pone.0223466)

Cluster no. 4

Go back to cluster table

Cluster is part of supercluster: 6

Cluster characteristics:

size	12605
size_real	15385
ecount	19984293
supercluster	6
annotations_summary	
pair_completeness	0.634962805526036
pbs_score	0
TR_score	0.685611111111111
TR_monomer_length	199
loop_index	0.97190030163518
satellite_probability	0.0194654605841841
consensus	ATTTTATACCATTTTGGACTCGTAAGGATCAGTACTATCGACTGGCATCAAAAAATAGAAAATCTAAATTTTGACCCAAA TCGACCAAAATGGCAAGGGGTTACATCACGATTTTTATCAAAATCGGGTTCGGTTCGAAAATACAACCTTTTTTGATGAT TTTTTTGAATATCTCGGCCAAATAGTGTCCGATTTTCAA
TAREAN_annotation	Putative satellite (low confidence)
orientation_score	1

Reads annotation summary

No similarity hits to repeat databases found

clusters with similarity:

Cluster	Number of similarity hits
11	290000
68	1900
36	1020
8	18
9	5
85	4
573	3
3	2
834	1

clusters connected through mates:

Cluster	Number of shared read pairs	k
11	2800	0.803
36	121	0.0613
68	60	0.0338
5	40	0.00467
85	21	0.012
8	11	0.00169
3	8	0.00226
1	5	0.00209
89	5	0.00283

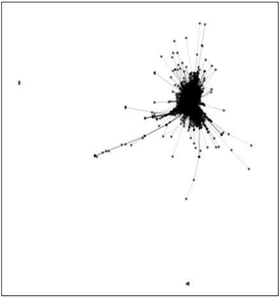

Supplement: S9 Fig — (PDF) [file pone.0223466.s009.pdf]
