# Supplementary material for: De novo identification of satellite DNAs in the sequenced genomes of Drosophila virilis and D. americana using the RepeatExplorer and TAREAN pipelines
Source: PLoS One. 2019 Dec 19;14(12):e0223466. doi: 10.1371/journal.pone.0223466 (PMC6922343; doi:10.1371/journal.pone.0223466)

Cluster no. 3

Go back to cluster table

Cluster is part of supercluster: 6

Cluster characteristics:

size	9210
size_real	9210
ecount	737297
supercluster	6
annotations_summary	
pair_completeness	0.86815415821501
pbs_score	0.1631687
TR_score	0.367599156926614
TR_monomer_length	171
loop_index	0.975895765472313
satellite_probability	0.751881405964912
consensus	AGACATAGTCAAAATTTCCCACCCCCATAACTCGGTCAAATCTCATCCGATTTTCACGAGGTTTGGCTTTTTGTTCATGG TTTTCCCTCTAGATTAATTTGGCATCAAAATCTGACAACATAATTTTTGGTCGAAATTCATGTCAAAATCTTACCCCAAG ATTCCTATAT
TAREAN_annotation	Putative satellite (high confidence)
orientation_score	1

Reads annotation summary

No similarity hits to repeat databases found

clusters connected through mates:

Cluster	Number of shared read pairs	k
1	31	0.013
44	13	0.0358
8	10	0.00386
2	7	0.00463
30	4	0.0106
3890	4	0.0122
11	3	0.00377
11500	3	0.00919
6	2	0.00155

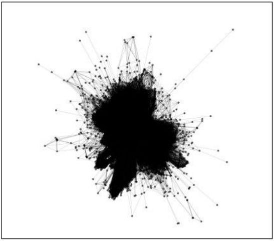

Supplement: S10 Fig — (PDF) [file pone.0223466.s010.pdf]
