# Supplementary material for: De novo identification of satellite DNAs in the sequenced genomes of Drosophila virilis and D. americana using the RepeatExplorer and TAREAN pipelines
Source: PLoS One. 2019 Dec 19;14(12):e0223466. doi: 10.1371/journal.pone.0223466 (PMC6922343; doi:10.1371/journal.pone.0223466)

Cluster no. 2

Go back to cluster table

Cluster is part of supercluster: 2

Cluster characteristics:

size	13646
size_real	25372
ecount	20004210
supercluster	2
annotations_summary	
pair_completeness	0.752209944751381
pbs_score	0
TR_score	0.58228
TR_monomer_length	171
loop_index	0.967457577315801
satellite_probability	0.0488727035644302
consensus	TAACTCGGTCAAATCTCATCCGATTTTCACGAAGTTTGGCTTTTTCTTCATGGTTTTCCCTCTAGATTAATCTGGCATTCAAATCTCGACACCGATTTTTTAGTCGAAATTCATGTCCAAATCTTACCCCAAGATTCGTATATAGACATGGGTCAAAAATTACCACCCCCA
TAREAN_annotation	Putative satellite (low confidence)
orientation_score	1

Reads annotation summary

No similarity hits to repeat databases found

clusters with similarity:

Cluster	Number of similarity hits
9	546000
5	3000
11	1460
8	458
202	50
656	20
36	5
20	3
5950	2

clusters connected through mates:

Cluster	Number of shared read pairs	k
9	1540	0.533
5	356	0.0412
8	180	0.0273
11	63	0.0177
36	23	0.0112
20	21	0.00795
1	16	0.00648
49	13	0.00662
656	10	0.00555

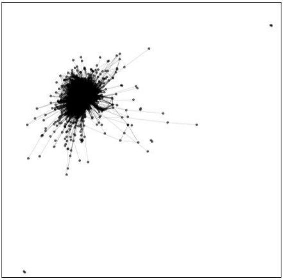

Supplement: S11 Fig — (PDF) [file pone.0223466.s011.pdf]
