# Supplementary material for: De novo identification of satellite DNAs in the sequenced genomes of Drosophila virilis and D. americana using the RepeatExplorer and TAREAN pipelines
Source: PLoS One. 2019 Dec 19;14(12):e0223466. doi: 10.1371/journal.pone.0223466 (PMC6922343; doi:10.1371/journal.pone.0223466)
Supplement: S12 Fig — (PDF) [file pone.0223466.s012.pdf]

Cluster no. 4

Go back to cluster table

Cluster is part of supercluster: 3

Cluster characteristics:

size	7182
size_real	7182
ecount	9236382
supercluster	3
annotations_summary	
pair_completeness	0.876175548589342
pbs_score	0
TR_score	0.814736591791589
TR_monomer_length	225
loop_index	0.994708994708995
satellite_probability	0.69915681403055
consensus	TTTAATAATACACTTTTATAACTTCATTATTCTTTTTATATAAACATAATTGACTTATGCCGGCAAGGCAACACTCCACA GTCATATGAATTTACCGTATTTAAAATTTTTCATATGACATGCCTTGCGCGACACCACTCCCTATATAGGTTTTTTGACA CATCATCTATAATGGCGATATTTTTTCCAAATGCTACCATTCACCTTAATGTCTTCATGTACTAA
TAREAN_annotation	Putative satellite (low confidence)
orientation_score	1

Reads annotation summary

No similarity hits to repeat databases found

clusters with similarity:

Cluster	Number of similarity hits
12	2060
150	19

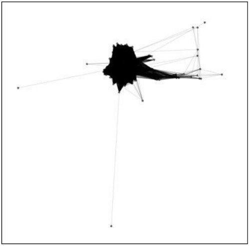

clusters connected through mates:

Cluster	Number of shared read pairs	k
12	168	0.4
1	31	0.0135
81	15	0.0591
2	8	0.00562
150	8	0.0322
9	4	0.00184
13	3	0.00548
505	3	0.0123
5	2	0.00168
