# Supplementary material for: De novo identification of satellite DNAs in the sequenced genomes of Drosophila virilis and D. americana using the RepeatExplorer and TAREAN pipelines
Source: PLoS One. 2019 Dec 19;14(12):e0223466. doi: 10.1371/journal.pone.0223466 (PMC6922343; doi:10.1371/journal.pone.0223466)

Cluster no. 7

Go back to cluster table

Cluster is part of supercluster: 3

Cluster characteristics:

size	10235
size_real	11853
ecount	19989815
supercluster	3
annotations_summary	
pair_completeness	0.861045690061234
pbs_score	0
TR_score	0.926558888888889
TR_monomer_length	225
loop_index	0.974694675134343
satellite_probability	0.767082439601018
consensus	ACATGCCTTGCTCGACACCACTCCCTATATAGGTTTTTTGACACGTCATCTATAATGGCGATATTTTTTCCAAATGCTAC CATTCACCTTAAGGTCATCATGTACTAATTTAATAATACATTTATATAACTTCATTATTCTTTTTATTTTAACATAATTG ACTTATGCCGGCAAGGCAACACTCCACAGTCATATGAATTTACCGTATTTAAAATTTTTCATATG
TAREAN_annotation	Putative satellite (high confidence)
orientation_score	1

Reads annotation summary

No similarity hits to repeat databases found

clusters with similarity:

Cluster	Number of similarity hits
12	43100
27	338

clusters connected through mates:

Cluster	Number of shared read pairs	k
12	686	0.809
27	166	0.295
304	16	0.0352
1	1	0.000896
4	1	0.000463
5	1	0.000137
11700	1	0.00225
24800	1	0.00225
28200	1	0.00225

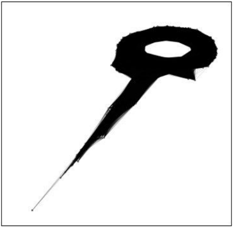

Supplement: S13 Fig — (PDF) [file pone.0223466.s013.pdf]
