# Supplementary material for: De novo identification of satellite DNAs in the sequenced genomes of Drosophila virilis and D. americana using the RepeatExplorer and TAREAN pipelines
Source: PLoS One. 2019 Dec 19;14(12):e0223466. doi: 10.1371/journal.pone.0223466 (PMC6922343; doi:10.1371/journal.pone.0223466)

# Cluster no. 6

Go back to cluster table

Cluster is part of supercluster: 4

## Cluster characteristics:

| | |
|---|---|
| size | 6233 |
| size_real | 6233 |
| ecount | 3935339 |
| supercluster | 4 |
| annotations_summary | |
| pair_completeness | 0.528070605540574 |
| pbs_score | 0 |
| TR_score | 0.428520374613522 |
| TR_monomer_length | 36 |
| loop_index | 0.948564158487823 |
| satellite_probability | 0.00706301117869965 |
| consensus | TAACTCCGCGCGGAGATATGACGTTCCAAAACGACA |
| TAREAN_annotation | Putative satellite (low confidence) |
| orientation_score | 0.999850127773719 |

**Reads annotation summary**

No similarity hits to repeat databases found

**clusters with similarity:**

| Cluster | Number of similarity hits |
|---|---|
| 46 | 31000 |
| 5 | 28100 |
| 87 | 738 |
| 130 | 355 |
| 26 | 275 |
| 91 | 55 |
| 9 | 48 |
| 182 | 23 |
| 707 | 6 |

**clusters connected through mates:**

| Cluster | Number of shared read pairs | k |
|---|---|---|
| 5 | 1240 | 0.648 |
| 8 | 61 | 0.0189 |
| 46 | 49 | 0.048 |
| 9 | 42 | 0.0145 |
| 24 | 30 | 0.0176 |
| 87 | 23 | 0.0229 |
| 1 | 19 | 0.0063 |
| 7 | 12 | 0.00756 |
| 142 | 8 | 0.00811 |

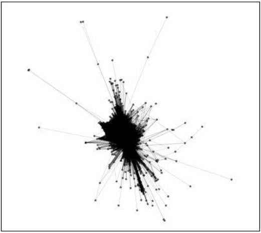

Supplement: S14 Fig — (PDF) [file pone.0223466.s014.pdf]
