# Supplementary material for: De novo identification of satellite DNAs in the sequenced genomes of Drosophila virilis and D. americana using the RepeatExplorer and TAREAN pipelines
Source: PLoS One. 2019 Dec 19;14(12):e0223466. doi: 10.1371/journal.pone.0223466 (PMC6922343; doi:10.1371/journal.pone.0223466)

Cluster no. 11

Go back to cluster table

Cluster is part of supercluster: 6

Cluster characteristics:

size	5811
size_real	5811
ecount	900900
supercluster	6
annotations_summary	
pair_completeness	0.244325481798715
pbs_score	None
TR_score	None
TR_monomer_length	None
loop_index	0.727491823033224
satellite_probability	0.000245279889944418
consensus	None
TAREAN_annotation	Other
orientation_score	0.999998890205845

Reads annotation summary

No similarity hits to repeat databases found

clusters with similarity:

Cluster	Number of similarity hits
4	290000
2	1460
36	592
5	32
85	2
9	1

clusters connected through mates:

Cluster	Number of shared read pairs	k
4	2800	0.803
36	143	0.0708
5	100	0.0116
2	63	0.0177
68	48	0.0263
8	29	0.00441
85	24	0.0133
9	16	0.00558
1	11	0.00451

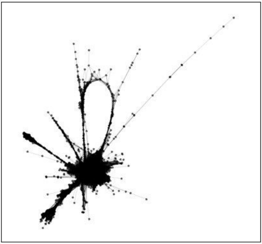

Supplement: S15 Fig — (PDF) [file pone.0223466.s015.pdf]
